# Does meaningful work mediate the relationship between empowering leadership and mental health? Evidence from Malaysian SME employees

**DOI:** 10.3389/fsoc.2023.1138536

**Published:** 2023-07-14

**Authors:** Muhammad Farhan Jalil, Bilal Tariq, Azlan Ali

**Affiliations:** ^1^School of Business and Management, University of Technology Sarawak, Sibu, Malaysia; ^2^Department of Economics, COMSATS University Islamabad, Vehari Campus, Vehari, Punjab, Pakistan; ^3^Graduate School of Management, Management and Science University, Shah Alam, Selangor Darul Ehsan, Malaysia

**Keywords:** empowering leadership, meaningful work, mental health, SMEs, structural equation modeling, Malaysia

## Abstract

**Introduction:**

In Malaysia, small and medium enterprises (SMEs) account for more than half of all employment and 98.7% of all businesses. There is little research on empowering behaviors in SMEs, despite leadership empowerment being often practiced. Therefore, the study aims to investigate how empowering leadership affects employees' mental health. The study also reveals meaningful work's role in mediating the relationship between empowering leadership and employees' mental health.

**Methods:**

A stratified random sample approach was used to collect data from 516 employees of Malaysian SMEs. The data was analyzed, and the hypothesis was tested using structural equation modeling (AMOS 21.0) with bootstrap confidence intervals computed to evaluate the mediating effect.

**Results:**

The results demonstrate that empowering leadership significantly improves employees' mental health. Furthermore, the association between empowering leadership and mental health is partially mediated by meaningful work.

**Discussion:**

This study contributes to the present empowering leadership-meaningful work-mental health model for SME employees, which reduces stress and anxiety at the workplace and positively impacts psychological empowerment and their capacity to control their overall emotions in instances of success.

## 1. Introduction

Work has taken on a more important role in our everyday lives, which has an impact on all aspects of a person's life, including their health (Kim et al., 2018). The health of employees, which includes both psychological and physical aspects, has a significant influence on an organization's performance (Salas-Vallina et al., 2021) and its ability to survive through impacting turnover (Bufquin et al., 2021), organizational citizenship behavior (Yu et al., 2021), absenteeism (Brunner et al., 2019), job performance (Tisu et al., 2020), and increasing medical and healthcare expenses (Song and Baicker, 2019). Beyond just having a financial impact, employment experiences may also influence parts of life outside of work (Akkermans et al., 2020).

Employee health policies and programs implemented by organizations are also seen as a sign that the company values its workers (Hu et al., 2021). Furthermore, it may help the company present the image of being an employer who cares about the wellbeing of its employees, which would attract recruits and keep a good workforce (Tripathi and Bharadwaja, 2020). The present study focuses on mental wellness and evaluates it based on small and medium enterprises (SMEs) employees' overall mental health.

According to research by Martin et al. (2009), < 25% of SME managers/owners had a clear policy on mental health, yet they felt that depression was a topic appropriate for discussion in the workplace. Lindström (2004) has suggested that “SMEs need special attention because their knowledge, competence, and financial resources to carry out interventions are limited” (p. 95). Stress management, mental health literacy, and employee support programs are examples of strategies that are often used by the leadership of the corporate sector but are challenging to execute and rarely used by SME owners or managers (De Angelis et al., 2020). Small businesses may provide a great environment for the implications of employee wellbeing, and it is a significant determining element in SMEs (Gerhardt et al., 2019). This is because SMEs have a limited number of employees and are usually closer to their leadership (owners/managers).

Leadership behavior is an important factor that can at least partially mitigate the detrimental effects of working circumstances on employees' attitudes (Farahnak et al., 2020). More specifically, leadership behavior plays a crucial role in creating a healthier working environment that has been positively linked to employees' wellbeing daily (Inceoglu et al., 2018). The importance of successful leadership behaviors for encouraging favorable employee attitudes has been thoroughly researched. Positively, the strongest protective factor for employees' mental health is empowering leadership behavior that shares power (Park et al., 2017).

Sharing power is a key component of empowering leadership to raise employee engagement and motivation (Alotaibi et al., 2020). It is vital to examine how constructive kinds of leadership, such as empowering leadership, affect the mental health of employees because of the critical role that leaders play in the social impact process inside businesses (Tripathi and Bharadwaja, 2020). Despite the growing body of research on the topic, very few researchers have focused on the effect of empowering leadership on employees' psychological health. Through studying the relationships between empowering leadership and employees' mental health, the current study aims to close this gap.

Recent research (such as Ghadi et al., 2013; Lee et al., 2017; Kim and Beehr, 2018) contends that the direct connection between empowering leadership and workplace psychology has varying intensities depending on the situation. They claim that such direct effects are not straightforward and might come about through assisting employees in understanding the meaningfulness of their work. The notion of meaningful work has been reported in many studies. As per Arnold et al. (2007), “meaningful work is all about finding a purpose in work that is greater than the extrinsic outcome of the work” (p. 195). According to Ghadi et al. (2013), employees' main objective is to be encouraged to search for work that is meaningful, rewarding, and inspiring. This viewpoint is clear in some of the earlier research on motivational theories (Kim and Beehr, 2018).

In contrast, according to Maslow's Hierarchy of Need, when one's basic needs for psychological support, physical safety, and social connection are addressed, one should work on higher-level desires, which include moving from “belonging” to “esteem” to “self-actualization” (Hale et al., 2019). It has been demonstrated that achieving these higher-level needs is intimately related to finding personal significance in one's employment (Ştefan et al., 2020). Once these demands are satisfied, people will look for a job that fulfills their life purpose, is more meaningful, and improves their psychological wellbeing (Ghadi et al., 2013). People, therefore, look for meaningful work that enhances their mental health (Allan et al., 2018). The relationship between meaningful work and mental health, Hackman and Oldham (1976) found that meaningful work, together with autonomy and feedback, improves mental health.

From this perspective, we contend that empowering leadership and meaningful work will be crucial to improving mental health. According to previous studies, SME employees' mental health is crucial due to their low compensation and rising healthcare costs (Cunningham et al., 2021; Park and Kim, 2021). Research on this subject in emerging economies has been encouraged since empowered leadership and meaningful work are important for SME employees. However, there has been a scarcity of research on the effects of empowering leadership and meaningful work on employees' mental health in the SME sector, particularly in Malaysia. Therefore, the main purpose of the study is to fill that gap.

Moreover, our research aims to empirically test the connection between empowering leadership and employee mental health as well as the mediating effect of meaningful work between them, both of which are lacking in prior research. Therefore, the following research questions are covered in this study;

*RQ1: Does empowering leadership have an impact on Malaysian SME employees' mental health?*
*RQ2: In Malaysia, does meaningful work mediate the association between empowering leadership and employee mental health?*

The following is a description of the paper's structure. Section 2 reviews the relevant literature, empirical studies, and develops hypotheses. Section 3 describe and design of research methodologies. Section 4 explain the findings of the analysis. Section 5 discusses the findings and conclusion of the study. Section 6 discuss implications of the study, and Section 7 represents limitations and recommendations.

## 2. Literature review and hypothesis development

### 2.1. Social exchange theory

According to Blau (1964), the social exchange theory describes how exchange interactions are sustained by the trustworthiness of rewards exchanged inside an organization. The idea of social exchange has undergone significant modification in a number of leadership studies (such as Settoon et al., 1996; Hooper and Martin, 2008; Eisenberger et al., 2014; Gooty and Yammarino, 2016). Employees respond positively to leadership acts that respect the reciprocity criterion since social exchange entails unclear commitments and future anticipated benefits (Gouldner, 1960; Blau, 1964). The theory contends that when owners or managers provide their employees with autonomy and assistance, the employees are more likely to show their appreciation for the owners or managers through their positive behavior and attitude. Employees who have developed strong bonds with their owners/managers (Kossek et al., 2011), for instance, frequently report higher levels of perceived social support, wellbeing, and productivity (Graen and Uhl-Bien, 1995; Eisenberger et al., 2014; Li and Liao, 2014).

This research contends that employees who perceive their leaders as helpful sources of power and resources are those who have been empowered by their leaders—SME owners or managers—through empowering activities. This claim is based on the social exchange theory's point of view. Employees who work for a reliable resource provider will consequently possess psychological inventiveness and a sense of support and value, which in turn causes them to feel satisfied and to give back by performing well in order to preserve positive working relationships with their managers/owners and firms.

### 2.2. Empowering leadership

According to Srivastava et al. (2006), empowering leadership is described as activities that share authority among team members and increase their intrinsic motivation levels. Empowering leaders display four sorts of behavior: they emphasize the importance of the task (Qian et al., 2018); allow for involvement in decision-making (Naqshbandi et al., 2018); exude confidence in the quality of performance (Huang et al., 2010); and remove any administrative restrictions (Amundsen and Martinsen, 2015). Based on the research of Conger and Kanungo (1988), these leadership empowerment techniques are not only about giving followers' power but also considered an important factor in motivating members. A team leader, therefore, must empower team members, include them in decision-making, have faith in their ability to enhance productivity, and make administrative regulations and processes simple in order to empower them and increase their motivation level (Park et al., 2017).

Recent empirical research has determined that empowering leadership has a positive association with organizational outcomes, including job satisfaction (Liu et al., 2021), task performance (Kundu et al., 2019), organizational citizenship behavior (Shahab et al., 2018), and commitment (Kim and Beehr, 2020). However, very little research has investigated how empowering leadership affects the psychology of employees at work. We also investigate the mediating effect of meaningful work between empowering leadership and workers' mental health because we believe it is crucial to recognize how leaders affect their employees' mental wellbeing.

### 2.3. Mental health

In the last few decades, the number of people suffering from mental illnesses has increased (Vuorre et al., 2021). According to Kessler et al. (2009), at least 18% of the world's population may experience mental illness at some point in their life. Individuals' mental health difficulties have been studied by psychiatric researchers. According to Kotera et al. (2019), poor mental health can lead to feelings of guilt and an incapacity to care for oneself or others.

According to Muris (2016), self-compassion is described as the understanding that mental illnesses are only human experiences, and it implies empathy for both oneself and those who suffer from mental illnesses. The study of Brouwers (2020) explains that people with mental illnesses are less efficient and interact less with their coworkers. Similarly, Joshi and Sharma (2020) describe how people who suffer from mental difficulties are more prone to losing their sense of self-worth and belonging. Furthermore, in a variety of business and personal contexts, people are regularly ostracized and isolated (Yang et al., 2022).

However, few developing countries have recognized the risks that individuals may face as a result of mental health issues (Yan et al., 2021). The Malaysian government recently unveiled its strategic psychiatric policy, which covers issues such as mental healthcare ease of access, endorsement of psychiatric disorder therapies, trying to prepare competent workers and instructors to deal with mental health issues (Ministry of Health, 2016), and establishing research institutes to conduct empirical studies on how to alleviate and build a resourceful frame against psychological illnesses (Mousa and Samara, 2022). Furthermore, a major topic that must be addressed here is how we can ensure that mental health help reaches individuals who need it (Torous et al., 2020), particularly in SMEs of underdeveloped countries where mental health diseases are ignored (Uzir et al., 2022). Finding solutions to avoid mental health difficulties becomes critical under such circumstances, especially when psychiatric therapy and knowledge are uncommon and not a concern for governments and businesses.

### 2.4. Meaningful work

The subjective perception that one's work is meaningful, promotes personal growth, and advances society is referred to as “meaning in work” (Allan et al., 2015). Finding meaning at work is regarded as a branch of meaning and serves as a prospective source of meaning in life (Steger and Dik, 2009; Ward and King, 2017; Zhang et al., 2019). Numerous studies (such as Ebersole and Devogler, 1981; Baum and Stewart, 1990; Emmons, 2005; Fegg et al., 2007) that queried participants about what gave their lives value and discovered similar replies, such as relationships, religion, service, and work, corroborate it. According to this view, experts contend that finding meaning at work leads to more meaning in life (Allan et al., 2015). This claim is backed up by several studies in which individuals regularly cite their jobs as a primary source of meaning (Ward and King, 2017).

Seligman (2002) has given attention to the concept of meaningful work as an area of positive psychology, which emphasizes the need to concentrate on actively cultivating the good elements of work and life. Rosso et al. (2010) defined meaningful work as work that is especially important and has greater personal significance. Asik-Dizdar and Esen (2016) stated that “the notion of meaningful work refers to a positive association between the individuals' participation and the rewarding results they obtain, such as happiness, efficacy, and contentment, among others” (p. 5). McConnell (2004, p. 14) define it as “the worth of a work aim or purpose, as measured against an individual's own beliefs or principles.”

Since it is strongly tied to employees' behavior and attitudes in the workplace, “meaningful work” has been acknowledged as a crucial term in research on vocational psychology (Ghadi et al., 2013). Previous studies have demonstrated a positive relationship between meaningful work and factors connected to the workplace, such as subjective wellbeing (Lintner and Elsen, 2018), job satisfaction (Ghislieri et al., 2019), and work engagement (Van Wingerden and Van der Stoep, 2018). These findings suggest that meaningful work is crucial for improving employees' mental health, both at work and in their personal lives.

Prior studies (such as, Martela and Pessi, 2018; Guo and Hou, 2022; Oprea et al., 2022) on the prerequisites of meaningful work either emphasized the features of the work directly or on individual work orientation. For instance, work significance, job enrichment, and person-job fit all support meaningful work. In terms of individual variables, emotional intelligence, work values, work volition, and work orientation all have an impact on meaningful work (Alotaibi et al., 2020).

Furthermore, it was found in recent research that leadership has a significant impact on how meaningful work is accomplished by employees (Ghadi et al., 2013). For instance, meaningful work is influenced by empowering leadership (Kim and Beehr, 2018), transformational leadership (Pradhan and Jena, 2019), and ethical leadership (Mostafa and Abed El-Motalib, 2020). As Lee et al. (2018) examined the effects of empowering leadership, LMX, and transformational leadership, they discovered that leaders' empowering behavior had the most influential impact on the psychological wellbeing of employees.

### 2.5. Dimensions of empowering leadership

Conger and Kanungo (1988) define empowerment as “a process of enhancing feelings of self-efficacy among organizational members through the identification of conditions that foster powerlessness and their removal by both formal organizational practices and informal techniques of providing efficacy information” (p. 474). Konczak et al. (2000) present the dimensions of empowering leadership: delegation of authority; accountability; self-directed decision making; information sharing; skill development; and coaching for innovative performance.

The study by Thomas and Velthouse (1990) identified that empowerment is a process that includes a supervisor sharing authority with employees. According to Conger and Kanungo's (1988) conception, empowerment denotes the distribution of power or delegation of authority, which should boost intrinsic motivation by altering task assessments pertaining to meaning, competence, self-determination, and influence (Burke, 1986). Therefore, Konczak et al. (2000) defined delegation of authority as one of the elements of empowering leadership. Therefore, we propose the following hypothesis:

*Hypothesis 1a. Delegation of authority is a significant component of empowering leadership*.

Konczak et al. (2000) explain one more component of empowering leadership as accountability, in which managers insist on outcomes accountability. Ford and Fottler (1995) claim that empowerment not only reallocates authority but also offers a way to hold team members accountable for achieving it. Conger (1989) shows how changes in power must be followed by a restructuring of performance assessment systems to ensure that individuals are evaluated and held responsible for performance they can influence. Therefore, we propose the following hypothesis:

*Hypothesis 1b. Accountability is a significant component of empowering leadership*.

Control, according to Tannenbaum (1986), is the capacity of the person to decide outcomes, behave as a causative agent, and also have an influence. The degree to which managers encourage autonomous decision-making should be a crucial component of the empowerment process since empowerment is linked to higher self-efficacy beliefs. Thus, Konczak et al. (2000) recognized encouragement of self-directed decision making as a component of empowering leadership. Therefore, we propose the following hypothesis:

*Hypothesis 1c. Self-directed decision making is a significant component of empowering leadership*.

Wellins et al. (1991) assert that in order to empower employees, managers must impart knowledge and information that will allow them to contribute as effectively as possible to the performance of the organization. Ford and Fottler (1995) explain that, instead of providing direction and control, the manager's role in skill development should be one of facilitation. A sizeable portion of the manager's time should be spent finding the right training to ensure that staff members acquire the skills necessary to support empowerment programs. Based on previous studies, information sharing and skill development were included by Konczak et al. (2000) as components of leader-empowering behavior. Therefore, we propose the following hypothesis:

*Hypothesis 1d. Information sharing is a significant component of empowering leadership*.
*Hypothesis 1e. Skill development is a significant component of empowering leadership*.

Another component of empowerment was identified by Konczak et al. (2000) and is referred to here as coaching for innovative performance. This aspect of empowerment involves leader behaviors that support calculated risk as well as innovation, offer performance feedback, and view failures and mistakes as moral lessons. Thomas and Velthouse (1990) noted that the word empowerment has become widely used at a time when organizations are looking for alternative management practices that foster dedication, risk-taking, and creativity due to international competition and change. While collaborating with subordinates to help them identify the causes of mistakes and lower the likelihood that they will occur again, leaders must make sure that taking risks is not penalized (Wallace, 1993; McConnell, 1994). Therefore, we propose the following hypothesis:

*Hypothesis 1f. Coaching for innovative performance is a significant component of empowering leadership*.

### 2.6. Empowering leadership and mental health

The psychological health of employees would be positively impacted by the empowering leadership style of SME owners/managers. In particular, leaders can have a beneficial impact on employees' psychological wellbeing when they adopt a participatory positive leadership attitude (Greenberg and Tracy, 2020). For instance, Gooty et al. (2009) find a similar positive relationship between transformational leadership's characteristics of empowerment and individual consideration and psychological wellbeing, and Rego et al. (2012) report a positive relationship between authentic leadership and psychological wellbeing.

Beneficial results are produced by empowering leadership that is characterized by positive leadership behaviors. Employees' psychological wellbeing is impacted by empowered leaders who use supportive actions to increase their intrinsic motivation (Suleman et al., 2021). For instance, previous research by Park et al. (2017) has shown that empowering leadership considerably decreases employees' levels of stress, anxiety, and depression and increases their sense of optimism for the future. Through allowing individuals to be independent in their work and encouraging them to adopt a meaningful attitude in the workplace, leaders' empowerment also helps employees become resilient (Avey et al., 2008). Indeed, it indicates that psychological wellbeing and a sense of meaning at work are significantly related to empowerment.

Avey (2014) notes a dearth of research on the effects on mental wellbeing and suggests that effective leadership (SME owners/managers) can have a significant effect on employees' mental wellbeing because these behaviors (such as encouraging others and removing obstacles) help employees to improve their mental health. Stuber et al. (2021) examine the effectiveness of leadership as a predictor of mental health in order to bolster his claim and find that it is the most effective predictor. To investigate the relationship between leaders' behaviors and employees' mental health, Walumbwa et al. (2010) argue that integrated research between mental health and effective leadership practices, such as empowering leadership, is needed. Based on previous research, we posit the following hypothesis:

*Hypothesis 1. Empowering leadership has a positive effect on the mental health of SME employees*.

### 2.7. Empowering leadership and meaningful work

According to Chalofsky (2003), workers who struggle to find purpose in their jobs include those who experience strong sentiments of rejection, bias, or misinterpretation. This study contends that by fostering perceptions of meaningful work, SME owners and managers can reduce or even eliminate such sentiments in an atmosphere where they exhibit empowering leadership behaviors. For instance, leaders may encourage their employees to be innovative and to find solutions to challenges by using knowledge-based motivation (Matsuo et al., 2019). The self-esteem of followers will increase in a setting where leaders are intellectually challenged. As a result, individuals do not even hesitate to voice their ideas in case they make a mistake for fear of being criticized (Kim and Beehr, 2018). This particular behavior may aid followers in managing their surroundings, which can prevent meaning in their job from emerging due to sentiments of rejection, discrimination, or misunderstanding (Matsuo et al., 2019). Scroggins (2008) stated that “consistency between work experiences and the individual's perception of self may enhance self-esteem, which will also make the work more meaningful” (p. 70).

Moreover, it may be argued that identifying meaningful work involves more than just the performance-related behaviors that employees have; it also involves the connection between purpose and values (Ghadi et al., 2013). SME owners or managers who create unique objectives, aims, and identities for businesses have the power to persuade employees that their work is meaningful (Martin et al., 2009). Empowering leaders are viewed as having a compelling future vision and conveying positivity about future objectives through inspiring motivation, which in turn strengthens followers' internal core values (Mendes and Stander, 2011). SME Owners' or managers' vision of the firm's mission and their subordinates' core values tend to align more frequently (Nanjundeswaraswamy, 2015). Therefore, subordinates are likely to consider the task to be more significant, motivating, and purposeful—all of which are essential elements in recognizing meaningful work (Ghadi et al., 2013). Hence, followers' view of meaningful work is likely to grow as owners or managers demonstrate more empowering leadership behaviors. Thus, we propose the following hypothesis:

*Hypothesis 2. Empowering leadership has a positive effect on the meaningful work of SME employees*.

### 2.8. Meaningful work and mental health

Work is meaningful, according to Steger et al. (2012), when it is regarded to be individually significant and contributes to a greater benefit. Meaningful work has been associated with benefits for both people and businesses in terms of an employee's mental health (Bufquin et al., 2021). Given the clear relevance of meaningful work to workers' happiness and favorable organizational outcomes (such as Kim and Beehr, 2018; Singh and Singh, 2018), the connection between meaningful work and health consequences has received little attention. Therefore, we bring up the research that shows meaningful work positively improves employees' mental health. In addition, Ryff and Singer (1998) advocated more “studies on how employment helps people find meaning in their lives, realize who they are, and use their unique talents, which in turn improves their health” (p. 8).

Burnout has been linked to strategies to improve meaningful work (Scanlan and Still, 2013), and meaningful work has been shown to mitigate the effects of job stress and improve the mental health of employees (Allan et al., 2018). The qualitative study of Mousa and Samara (2022) revealed that employees who can do meaningful work may experience less stress and have better mental health. Considering the nature of SME employees, who are needed to handle a heavy workload (Vanharanta et al., 2022), work overtime, and perform other admin duties, the participants claimed that their job duties caused them to experience constant depression, anxiety, stress, and other mental disorders (Chhinzer, 2022). Although meaningful work appears to be a protective factor, no research has been done to investigate if it may mitigate the impacts of stress and enhance the mental health of SME employees. Thus, we believe that meaningful work can have an influence on SME employees' mental health, and this study proposes the following hypotheses:

*Hypothesis 3. Meaningful work has a positive effect on the mental health of SME employees*.

### 2.9. Mediating effect of meaningful work

The prime objective of meaningful work has been the supposition that perceptions of meaningful work are related to personal and organizational outcomes (Ghadi et al., 2013). The contribution of this research lies in its investigation of how the relationship between empowering leadership and mental health is mediated by perceptions of meaningful work. The concept of meaningful work has also been identified in important models, like the empowerment model (Jena et al., 2019) and the model of job characteristics (Simonet and Castille, 2020), which provides more evidence of the significance of meaningful work.

However, the significant improvements that have taken place over the past two decades, including demographic shifts, globalization, and technological advancement, have had an impact on employee behavior and views of work (Ghadi et al., 2013). Therefore, Rosso et al. (2010) point out that scholars need to be more specific about the kind of meaningful work they do, as older techniques to determine meaning may no longer be suitable in light of these current developments. In fact, Scroggins (2008) asserts that despite the meaningful work appearing in a number of models, the construct in the literature on organizational behavior has only lately attracted attention. Because different conceptualizations in the literature have led to problems with the construct's growth, scholars should be more selective about the kind of meaningful work they are addressing (Pratt and Ashforth, 2003; May et al., 2004).

This research assessed some of the significant studies that looked at the importance of meaningful work in recent years to solve these concerns. From this assessment, this study can assume that employees' views of meaningful work are mostly reliant on interactions and subjective assessments of their work environments. The study further implies that workers perceive meaningful work when it has a goal, purpose, and value related to the employee and his/her capacity to create meaningful work, as well as when there is an interaction between the values and goals of the employees and those of the organization and the workplace (Rosso et al., 2010).

In this study, the relationship between empowering leadership and mental health is considered to be mediated by meaningful work. Baron and Kenny (1986) outlined two prerequisites: first, empowering leadership needs to be connected to both meaningful work and mental health; and second, the initial relationship between empowering leadership and employees' mental health must be reduced by including meaningful work in the analysis. The only method to determine the mediating effect when both requirements are satisfied is to do a statistical analysis of them. This study has previously evaluated the pertinent literature on the connection between mental health, empowering leadership, and meaningful work. Therefore, this study proposes the following hypotheses:

*Hypothesis 4. The mediating effect of meaningful work between empowering leadership and the mental health of SME employees*.

### 2.10. Hypothesized framework

**Figure 1:**
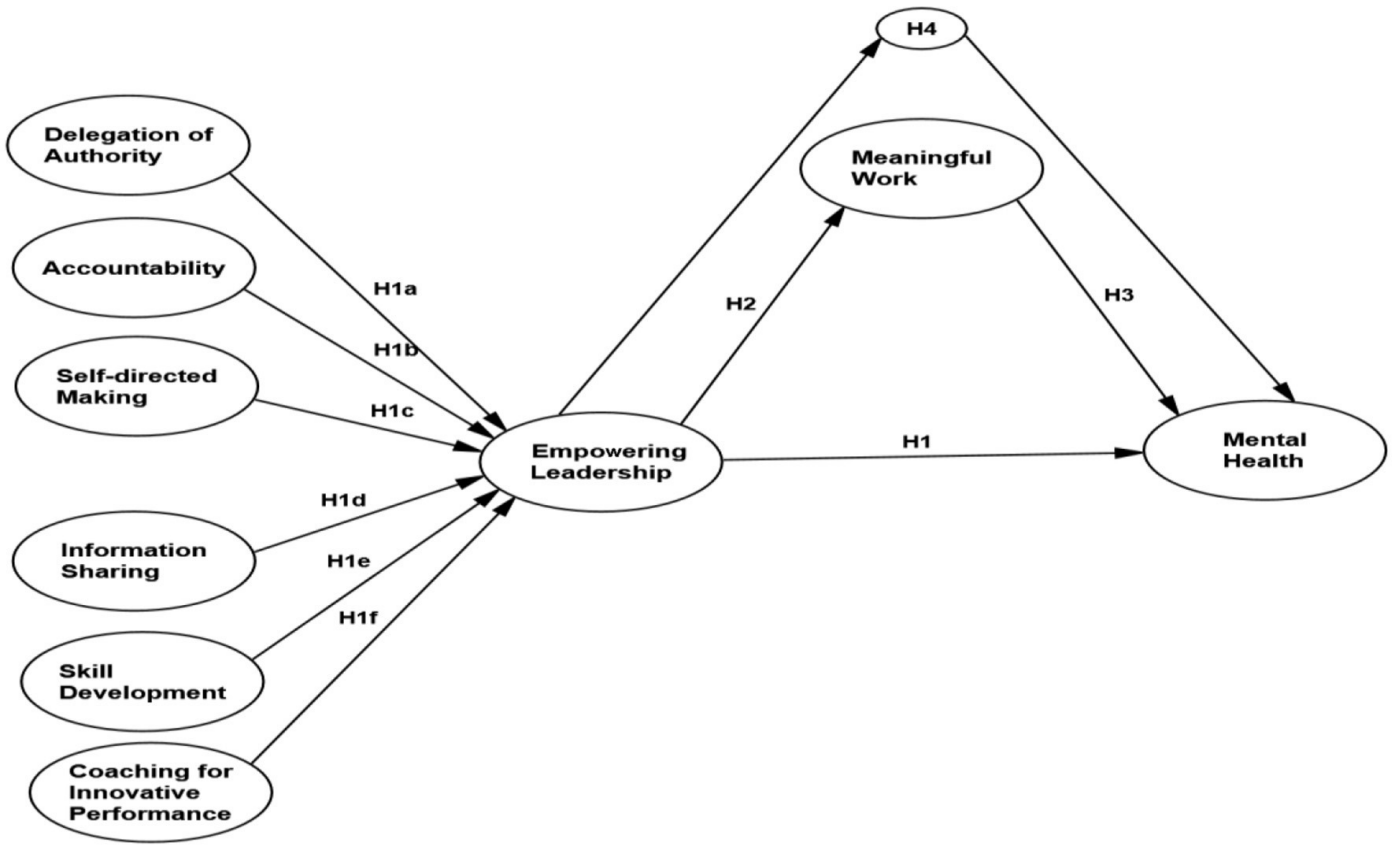
Hypothesized framework.

Previous studies (Amundsen and Martinsen, 2015; Alotaibi et al., 2020; Kim and Beehr, 2020) have examined the influence of empowering leadership on job satisfaction, organizational commitment, and work engagement. Few studies have considered the impact of empowering leadership on employees' mental health in SMEs. Therefore, the specific objectives of the study are: first, to identify the effect of empowering leadership on employees' mental health; second, to assess the mediating impact of meaningful work on the mental health of SME employees. The hypothesized framework is shown in Figure 1.

## 3. Methods

### 3.1. Specification of micro, small, and medium enterprises

The definition of micro, small, and medium enterprises (MSMEs) varies from country to country. The definitions of MSMEs used in this study are shown in Table 1 by SME Crop Malaysia.

**Table 1:** Classification adopted by SME Crop Malaysia.

**Size category**	**Employment**	**Assets (RM million)**
Micro enterprises	< 5 employees	< RM 0.25
Small enterprises	Between 5 and 50 employees	Between RM 0.25 and < RM 10
Medium enterprises	Between 51 and 150 employees	Between RM 10 and < RM 25

### 3.2. Data collection: procedure and sample

We used a survey approach in order to obtain data from participants in Malaysia. Furthermore, in this study, data was collected using a close-ended structural questionnaire through a self-administered and online approach.

The sample size within that target audience must be specified for the study (MacCallum et al., 1999). In the same line, according to Schreiber et al. (2006), the sample size necessary for structural equation modeling (SEM) should be at least 200. Respondents in this study were SME employees, and data was obtained from six different Malaysian states: Kedah, Selangor, Sarawak, Sabah, Penang, and Johor. The questionnaires were sent to a total of 610 respondents using a stratified sampling method.

This sampling method divides the population into two or more significant and pertinent strata when the population is diverse in the variables or traits under consideration (Tong, 2006). As a result, only 516 questionnaires were completed, yielding a response rate of 84.6%.

### 3.3. Measurement of variables

According to Hair et al. (2014), the survey questionnaire was designed to collect the information necessary to address the research questions and meet the key goals of the study. The data for this study was acquired using adapted items from previous studies in order to assess the influence of meaningful work on SME employees' mental health through coping strategies. To allow participants to focus on the questions, the items in the questionnaire were evaluated on a 7-point Likert scale. To measure empowering leadership, the items were adapted from Konczak et al. (2000). To measure mental health, items were adapted from Hu et al. (2007) and Mazaherinezhad et al. (2021). The items were adapted from Steger et al. (2012) to measure meaningful work.

### 3.4. Ethical consideration

The University of Technology Sarawak Ethics Committee (UTS-EC) authorized the procedure (The Declaration of Helsinki) after all research participants provided written consent. The data was then processed and forwarded to be analyzed further.

### 3.5. Statistical analysis

The acquired data was separated into two parts for analysis in this study. The Statistical Package for Social Sciences (SPSS) 22.0 version was used in the first part for descriptive statistics about the respondents and preliminary data analysis. The analysis of moment structures (AMOS) 21.0 version was employed to screen and investigate the associations among constructs within the suggested conceptual framework during the second stage of SEM.

Due to its widespread acceptability among academic researchers, SEM, which is also known as path analysis, is used to evaluate and assess the hypothesized correlations among numerous independent and dependent constructs at the same time inside the suggested framework (Kline, 2010; Ryan, 2018). Hair et al. (2010) stated that “there are six steps in the SEM decision process; first: Defining individual constructs, second: Developing the overall measurement model, third: Designing a study to produce empirical results, fourth: Assessing measurement model validity, fifth: Specifying the structural model, and sixth: Assessing structural model validity” (p. 654).

Within the provided conceptual framework, this study gives an in-depth investigation of the correlations among the variables. During the data analysis, two stages were employed. The confirmatory factor analysis (CFA) was used in the first phase to examine the components' validity and fitness in the model. The hypothesized associations between the variables (independent and dependent) were then tested using the structural model approach. When using the SEM two-step approach, according to Hair et al. (2010), only those items that have excellent measurements (validity and reliability) will be incorporated into the structural model.

## 4. Results

### 4.1. Demographic characteristics

In this study, demographic information was gathered from 516 employees working in 302 randomly chosen SMEs. Table 2 displays the demographic information such as gender, age, marital status, education, ethnic group, religion, enterprise level, enterprise activities, position in the enterprise, and income level. Moreover, the demographic information, particularly the range between age and income, was used from the SME Crop Malaysia.

**Table 2:** Demographic profile of respondents.

**Constructs**		**Number**	**Percentage**
Gender	Male	337	65.3%
	Female	179	34.7%
Age	Below 25	42	8.1%
	25–35	113	21.9%
	36–45	157	30.4%
	46–55	173	33.6%
	55 and above	31	6.0%
Marital status	Single	82	15.9%
	Married	377	73.1%
	Widow	21	4.1%
	Divorced	36	6.9%
Education	Diploma or high school or less	167	32.4%
	Bachelors	241	46.7%
	Masters	87	16.9%
	Doctorate	21	4.0%
Ethnic group	Malay	252	48.8%
	Chinese	159	30.8%
	Indians	76	14.7%
	Others	29	5.7%
Religion	Muslim	261	50.7%
	Hindu	44	8.5%
	Christian	103	20.0%
	Buddhist	87	16.7%
	Others	21	4.1%
Enterprises level (based on number of employees, *N* = 302)	Micro and small enterprise	179	59.3
	Medium enterprise	123	40.7
Enterprises activities (*N* = 213)	Manufacturing sector	96	31.8%
	Services sector	206	68.2%
Position in the enterprise	Lower level executives	108	20.9%
	Middle level executives	287	55.7%
	Upper level executives	121	23.4%
Income level	< RM3000	58	11.2%
	RM 3,000–4,000	246	47.7%
	RM 4,001–5,000	109	21.1%
	RM 5,001 and above	103	20.0%

*N*, number of selected SMEs.

### 4.2. Normality statistics

According to Yun et al. (2019), in multivariate analysis, normality must be tested. They further explain that, if the dataset is indeed not normally distributed, the validity and reliability of the findings may be compromised.

The Skewness-Kurtosis test was used in this research to determine whether the data was normally distributed. According to Pallant (2010), the value of skewness and kurtosis show the distribution's homogeneity. Furthermore, Tabachnick et al. (2007) identified that the usual range for skewness-kurtosis value is ±3. In response to a suggestion, Table 3 shows that all of the constructs in this research were determined to be normally distributed (i.e., ±3).

**Table 3:** Descriptive statistics.

**Varibales**	**Likert scale**	**Mean**	**Std. Dev**.	**Skewness**	**Kurtosis**
Delegation of authority	1–7	4.68	0.34	0.107	−0.098
Accountability	1–7	4.95	0.39	0.065	0.462
Self-directed decision making	1–7	5.77	0.43	0.246	0.076
Information sharing	1–7	6.23	0.49	0.083	−0.082
Skill development	1–7	5.41	0.38	0.186	0.543
Coaching for innovative performance	1–7	5.29	0.44	0.382	0.188
Meaningful work	1–7	6.13	0.52	−0.045	0.076
Mental health	1–7	5.86	0.59	−0.871	−0.021

### 4.3. Reliability and correlation of the constructs

According to Heale and Twycross (2015), the consistency of a measure utilized in the research is referred to as reliability. Furthermore, they explain that when we perform the same study with other samples and give the identical initial circumstances for the test, we may call it reliable.

Coefficient alpha was used to test the constructs' reliability in this study (Cronbach, 1951). Coefficient alpha is a metric that assesses how effectively a group of items measures a single unidirectional latent concept (Kost and da Rosa, 2018). Different researchers thought different dependability levels were adequate. According to DeVellis and Thorpe (2021), it should be at least 0.7, with a value of 0.8 or higher being ideal. In other words, if Cronbach's coefficient approaches 1.0, the constructions are very reliable. The reliability tests in this research were analyzed using SPSS, as shown in Table 4.

**Table 4:** The reliability correlation of the constructs.

	**Alpha**	**1**	**2**	**3**	**4**	**5**	**6**	**7**	**8**
Delegation of authority	0.934	1							
Accountability	0.965	0.331	1						
Self-directed decision making	0.947	0.329	0.372	1					
Information sharing	0.943	0.358	0.392	0.317	1				
Skill development	0.921	0.421	0.396	0.383	0.433	1			
Coaching for innovative performance	0.952	0.345	0.332	0.448	0.378	0.487	1		
Meaningful work	0.878	0.489	0.427	0.476	0.465	0.448	0.459	1	
Mental health	0.896	0.541	0.492	0.485	0.476	0.413	0.469	0.422	1

Pearson's correlation is used to assess the correlation between different variables in this research. The direction and magnitude of the linear association between the variables may be calculated using correlation coefficients (Armstrong, 2019). According to Benesty et al. (2009), “Pearson's correlation coefficients (*r*) indicate whether there is a positive or negative association and range from −1 to +1” (p. 5). Furthermore, Ahlgren et al. (2003) explain that the magnitude of absolute value provides information on the relationship's strength. Table 4 summarizes and presents the Cronbach's alpha findings as well as correlations between the variables.

### 4.4. Analysis of measurement model

**Figure 2:**
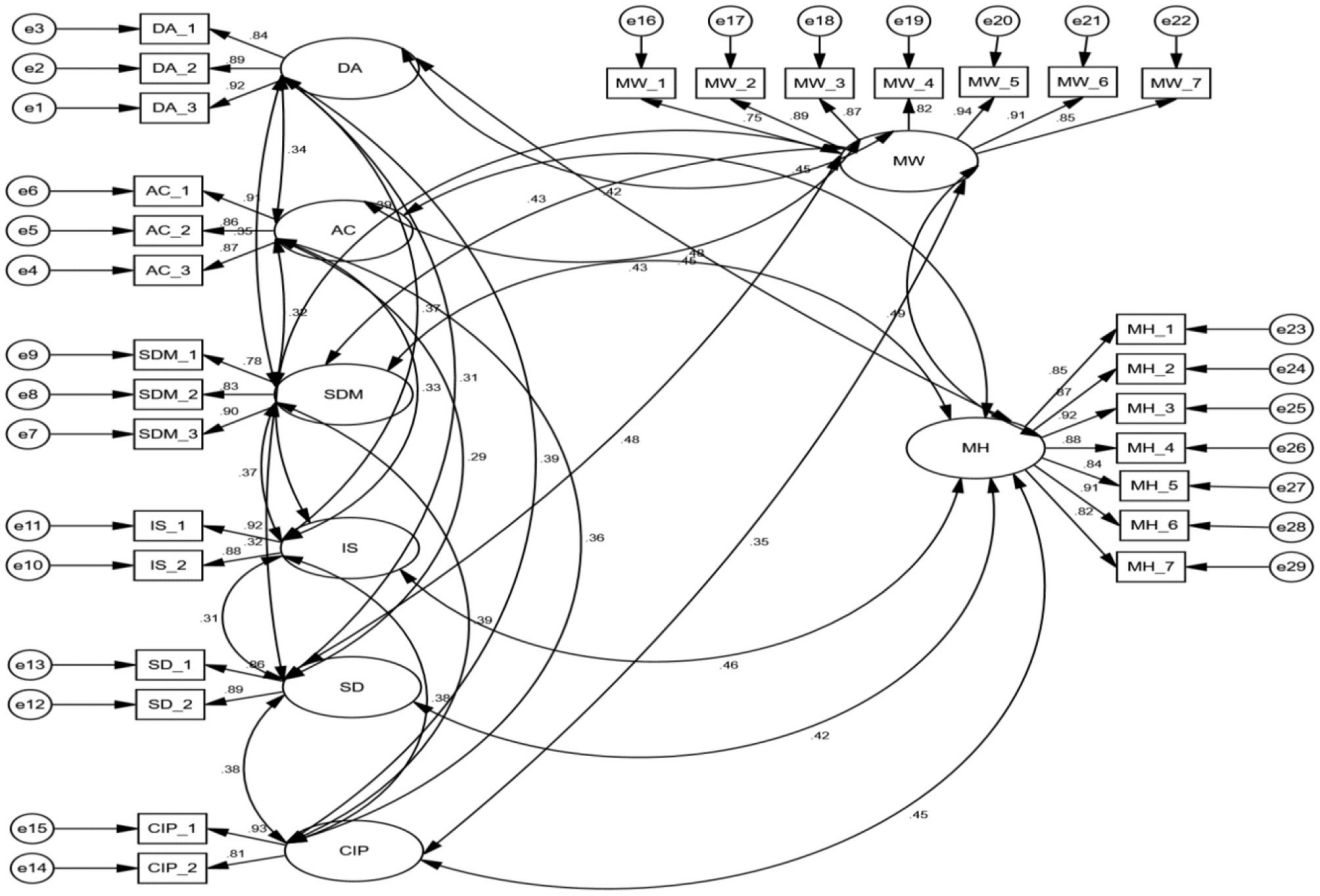
Measurement model. DA, delegation of authority; AC, accountability; SDM, self-directed making; IS, information sharing; SD, skill development; CIP, coaching for innovative performance; MW, meaningful work; MH, mental health.

Confirmatory factor analysis (CFA) was used in this study to evaluate the links between the variables studied in the conceptual model. In order to evaluate the measurement model in CFA, the researcher first assessed the measurement model fit before evaluating the measurement model validity (Marsh et al., 2009). As shown in Figure 2, the measurement model reveals that the factor loading of each item is adequate (above 0.70) as identified by Klein et al. (2001). To estimate the model's parameters, the researchers used the maximum-likelihood technique, with all analyses based on variance-covariance matrices. In order to measure the model's goodness-of-fit, various fit indices should be examined (Hair et al., 2010).

In the examination of overall measurement model, modification indices indicated that the indicators IS_3 (information sharing), SD_3 (skill development) and CIP_3 (coaching for innovative performance) had unacceptably high values. After iteratively removing these redundant items, the overall model fitness came up in good shape.

The model goodness-of-fit in this research was in the acceptable range (RMSEA = 0.041; chi square = 528.973; df = 515; GFI = 0.932; AGFI = 0.947; CFI = 0.951; CMIN/df = 1.566).

Examining the validity and reliability of the measurements is an essential process before testing the hypotheses in the suggested conceptual framework, since this may impact the results and hence the study objectives (Alias et al., 2015). Hair et al. (2010) determined that validity and reliability may be determined using “Composite Reliability (CR) and Average Variance Extracted (AVE)”. Furthermore, they recommend that CR should be more than 0.6 and ideally over 0.7 in order to demonstrate dependability, and the AVE should be >0.5 to show convergent validity. The AVE for all variables in this study was >0.5, and the CR was >0.7. As indicated in Table 5, all components have good reliability and convergent validity.

**Table 5:** AVE and CR evaluation.

**Items**	**Measurement path**	**FL**	**CR**	**AVE**
**Delegation of authority**				
DA_1	My manager/owner has given me authority to decide how to enhance the work process and procedures.	0.84	0.915	0.781
DA_2	My manager/owner gives me the power to implement the changes required to make things even better.	0.89		
DA_3	My manager/owner gives me the same level of authority that I am given in terms of responsibility.	0.92		
**Accountability**				
AC_1	I am considered accountable for the task given by my manager or owner.	0.91	0.912	0.775
AC_2	I am accountable for my performance and outcomes.	0.86		
AC_3	My manager/owner holds staff in the department accountable for maintaining customer satisfaction.	0.87		
**Self-directed decision making**				
SDM_1	When an issue arises, my manager/owner seeks to encourage me to find my own solutions rather than dictate what he or she would do.	0.78	0.876	0.702
SDM_2	My manager/owner depends on me to make independent decisions about matters that have an impact on how work is completed.	0.83		
SDM_3	My manager/owner pushes me to come up with my own solutions to issues I encounter at work.	0.90		
**Information sharing**				
IS_1	My manager/owner is responsible for sharing the knowledge or information that I need to produce significant results.	0.92	0.895	0.810
IS_2	I receive the information I require from my manager/owner to satisfy the needs of my consumers.	0.88		
**Skill development**				
SD_1	My manager/owner frequently gives me the chance to learn new skills.	0.86	0.867	0.766
SD_2	Continuous learning and skill development are priorities in our department, and my manager/owner makes sure of this.	0.89		
**Coaching for innovative performance**				
CIP_1	I'm encouraged to try out novel ideas, even if there is a possibility they won't work.	0.93	0.863	0.761
CIP_2	When I make a mistake, my manager/owner focuses on taking corrective action instead of placing blame.	0.81		
**Meaningful work**				
MW_1	I understand how my work adds to the meaning of my life.	0.75	0.953	0.745
MW_2	I have such a strong understanding of what makes my work meaningful.	0.89		
MW_3	I view my work as contributing to my own progress.	0.87		
MW_4	My work assists in my understanding of myself.	0.82		
MW_5	My work helps me make sense of the world around me.	0.94		
MW_6	My work has a significant impact on the organization.	0.91		
MW_7	The work I undertake has a larger meaning.	0.85		
**Mental health**				
MH_1	I am able to focus on my work.	0.85	0.956	0.758
MH_2	I did not lose much sleep because of anxiety.	0.87		
MH_3	I did not feel overworked.	0.92		
MH_4	I am optimistic that I will be able to tackle my work-related challenges.	0.88		
MH_5	I do not feel unhappy or depressed.	0.84		
MH_6	Recently, I've begun to believe in myself.	0.91		
MH_7	I consider myself to be a valuable individual.	0.82		

### 4.5. Assessment of structural model

**Figure 3:**
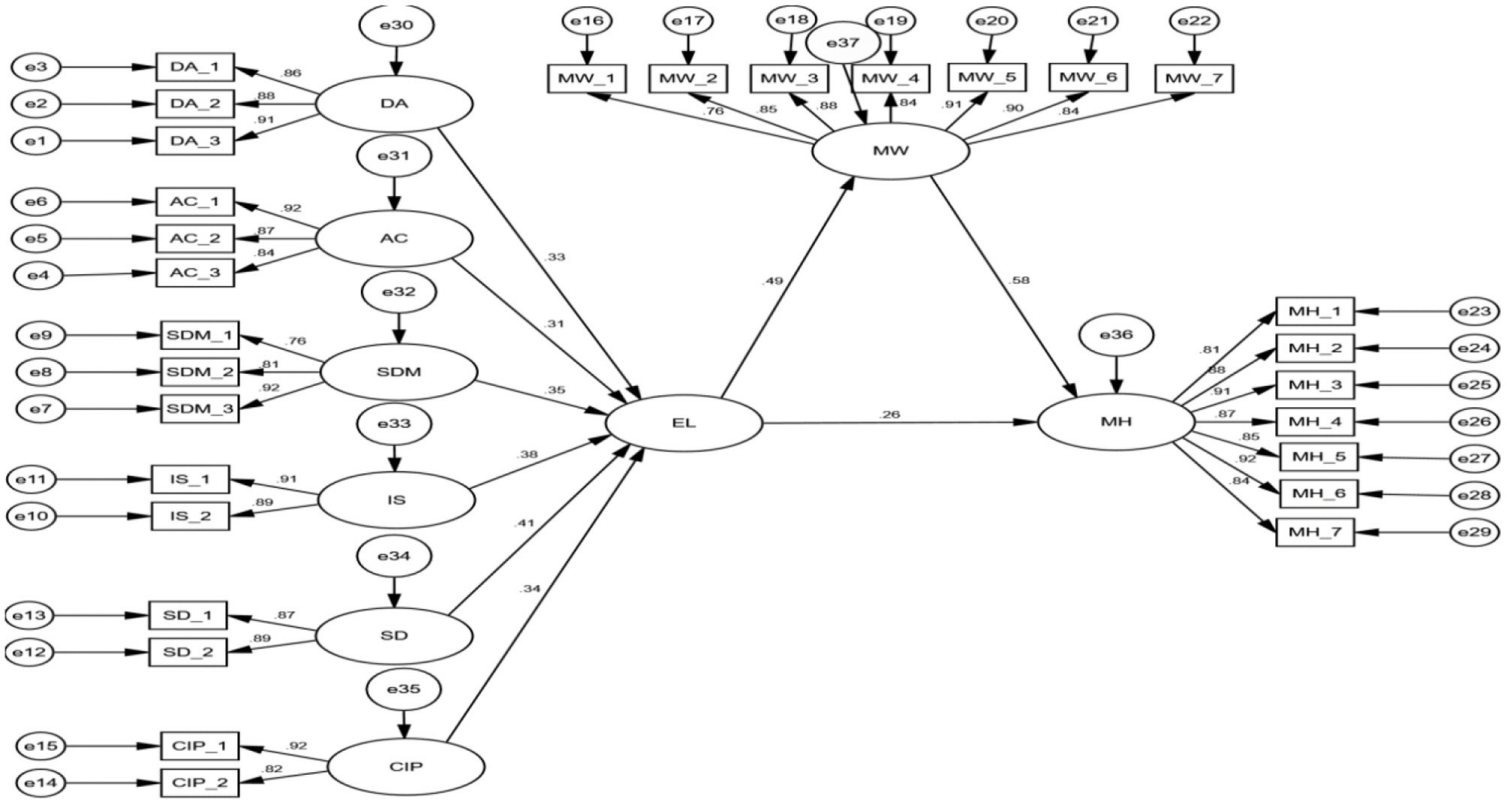
Structural model.

After the evaluation of CR and AVE, Boker et al. (2011) recommend testing the associations among exogenous and endogenous latent constructs, which can be performed at the structural model stage. According to Iasiello et al. (2022), it is essential to determine the goodness-of-fit for the structural model. The fit indices were (Chi-square = 1.298; GFI = 0.946; AGFI = 0.935; CFI = 0.956; TLI = 0.961; RMSEA = 0.039), suggesting that the model is well-fitted, as shown in Figure 3.

The next step in this research is to examine the model's hypothesized relationships. The hypotheses “H1a, H1b, H1c, H1, H2, H3, H4, and H5”, as shown in Table 6, were significant in assessing the hypothesized links within the suggested research model.

**Table 6:** Testing direct relationship.

**Hypothese and paths**	**ß**	***Z*-value**	**Supported**
H1a: Delegation of authority –> Empowering leadership	0.33^***^	4.034	Yes
H1b: Accountability –> Empowering leadership	0.31^***^	3.989	Yes
H1c: Self-directed decision making –> Empowering leadership	0.35^***^	4.207	Yes
H1d: Information sharing –> Empowering leadership	0.38^***^	4.519	Yes
H1e: Skill development –> Empowering leadership	0.41^***^	4.698	Yes
H1f: Coaching for innovative performance –> Empowering leadership	0.34^***^	4.117	Yes
H1: Empowering leadership –> Mental health	0.26^**^	3.758	Yes
H2: Empowering leadership –> Meaningful work	0.49^***^	4.996	Yes
H3: Meaningful work –> Mental health	0.58^***^	5.767	Yes

^*^p < 0.05, ^**^p < 0.01, ^***^p < 0.001.

### 4.6. The mediation analysis

The research hypothesis 4 is to examine whether meaningful work mediates the association between empowering leadership and the mental health of SME employees. According to Baron and Kenny (1986), once the direct effect and indirect effect point in the same direction, it is considered partial mediation. According to the findings, the indirect effect of meaningful work between empowering leadership and mental health was 0.28 (0.49 × 0.58 = 0.28), while the direct effect was 0.26. As Jalil et al. (2021) revealed, this study also used the bootstrapping approach to confirm the study's findings, which suggested that partial mediation had occurred, as shown in Table 7.

**Table 7:** Bootstrapping results.

**Paths**	**Relationship**	**Mediation**
Empowering leadership –> Mental health	0.27	
Empowering leadership –> Meaningful work –> Mental health	0.50 × 0.59 = 0.30	Partial

## 5. Discussion

By using social exchange theory (Blau, 1964), this study has demonstrated how empowering leadership affects workers' mental health. This research examined specifically how empowering leadership improves the mental health of employees and how meaningful work mediates this relationship. The findings of the study indicated that empowering leadership by SME owners and managers significantly improved the mental health of employees. Additionally, it was shown that meaningful work had a favorable relationship with both empowering leadership and employee mental health. Furthermore, the connection between empowering leadership and employees' mental health is partially mediated by the effect of meaningful work.

The demographic findings of the study show that male employees with an age group between 35 and 55 years, holding a bachelor's degree, belonging to the Malay ethnic group, working in micro and small enterprises, particularly in the services sector, with an income level between RM 3,000 and 4,000, express the concern and importance of empowering leadership toward mental health in the workplace.

Despite the limited amount of prior research on empowering leadership, it shows that the concept deserves additional study. To further advance this research, the findings identified empowering leadership components, including “delegation of authority, accountability, self-directed decision making, information sharing, skill development, and coaching for innovative performance” that help to improve the psychological state of employees. The findings are supported by the earlier studies of Konczak et al. (2000) and Alotaibi et al. (2020), which found that factors of empowering leadership such as “delegation of authority, accountability, self-directed decision making, information sharing, skill development, and coaching for innovative performance” had a substantial impact on psychological wellbeing. It also implies that all these components are vital, and that portraying each dimension could be incredibly beneficial in practical work with individuals, assisting in the identification of specific satisfactions and inadequacies related to a person's job experience.

The primary aim of this research was to evaluate whether or not empowering leadership has a significant impact on the mental health of SME employees. We found that empowering leadership improves employees' mental health, directly supporting our hypotheses. This is consistent with recent research by Park et al. (2017), Kim et al. (2018), and Tripathi and Bharadwaja (2020), which suggests that empowering leadership is beneficial for employees' psychological wellbeing.

According to Ghadi et al. (2013), the relationship between leadership and psychological wellness has been found to be significantly mediated by meaningful work. The study's findings confirmed that meaningful work has a partial mediating impact on the connection between empowering leadership and employees' mental health in SMEs. The study contends that employing a meaningful work strategy and empowering leadership (in this case, SME owners and managers) can improve employees' mental health. The results of the research are consistent with Matsuo et al. (2019) research by indicating that meaningful work has an indirect influence on empowering leadership and employees' psychological wellbeing.

This study looks at a conceptual model that incorporates the body of knowledge on empowering leadership toward the psychological wellbeing of employees. In doing so, it makes an effort to combine employee participation with empowerment from small business owners and managers. Furthermore, the research exclusively focuses on SME employees in Malaysia, where there are few studies on psychological health, which is quite different from that of the corporate sector. The vocational psychological health literature might benefit from an understanding of these connections in the context of Malaysian SME employees.

## 6. Theoretical and practical implications

### 6.1. Theoretical implications

The study has several theoretical implications. The first theoretical contribution of this research concerns the effect of empowering leadership on the mental health of SME employees. After carefully considering the social exchange theory, the authors of this study chose to adopt a quantitative research approach, including a survey questionnaire, to get the required data from SME employees in order to acquire more in-depth insights into the mechanism by which this phenomenon occurs. The authors concluded from their examination of the data that empowering leadership (SME owners/managers) can improve the mental health conditions of employees in Malaysia. Based on the findings, we believe that empowered SME leaders can minimize any unfavorable mental health conditions.

Previous studies have not assessed how meaningful work links empowering leadership with SME employees' mental health. Therefore, the study's second theoretical contribution is to demonstrate empirically how SME employees may enhance their mental health by using meaningful work driven by empowering leadership. According to the findings, empowering leadership assists in the building of mental health in order to recover and survive psychology-related challenges as well as the adoption of meaningful work to improve their relationship.

The third major theoretical contribution to social exchange theory is demonstrating empirically that empowering leadership of SMEs should pay close attention to their employees' meaningful work in order to maintain a semblance of intent for their responsibilities and thus alleviate any mental illnesses. This might lead to other researchers reconsidering social exchange theory and assessing the effects of using it in other sectors.

### 6.2. Practical implications

This research showed that in order to increase employees' psychological wellbeing, they must emphasize meaningful work and, for that reason, empowering leadership, which plays an important role in improving the employees' mental health. Therefore, SME owners or managers should foster a sense of empowerment in their employees by allowing them to take on more difficult but meaningful tasks, increasing their feelings of competence, giving them more decision-making authority at work, and providing them with the opportunity to exercise influence by engaging them in strategic goal-setting and making job outcomes recognizable and beneficial.

The research's findings have significance for Malaysian SME managers and owners in terms of empowerment initiatives. For psychological wellbeing, it is essential to create employment that allows for self-determination and has personal significance for the employees. SME processes need to be simplified in order to improve the employees' impression of their work's significance, competence, autonomy, and influence. The development of empowering work environments may be greatly influenced by SME owners and managers. They may encourage a better work environment by responding to employee input, providing greater power, and promoting self-initiative. Owners and managers must promote employee involvement and voice from middle or lower management in order to achieve true empowerment.

## 7. Limitations and recommendations

We should acknowledge the limitations of this study. This study's data sample covers executives from lower, middle, and upper levels of management. The degree of empowerment or the scope of the impact of empowering leadership may also rely on the respondent's organizational structure if we believe that delegation typically follows a hierarchy and proceeds top-down. Therefore, comparing the disparities between non-professional workers and professionals in terms of the impacts of empowering leadership would be valuable for future study.

Future studies might focus on SME owners and managers to learn about their perspectives on the problems they encounter and also strategies to alleviate anxiety, depression, and some other mental health issues. Furthermore, the authors encourage other academics from the field of vocational psychology to participate in multi-disciplinary scholarly articles aimed at identifying the primary determinants that may positively affect employees' mental health.

Moreover, we recommend more research that examines the impact of other leadership styles in contrast to empowering leadership when looking at employees' mental health. “Authentic leadership, ethical leadership, and empowering leadership” were the three positive leadership styles that Avey (2014) tested as antecedents of psychological wellbeing. However, there are other domains left out in those leadership styles, particularly regarding servant and spiritual behaviors. Future studies on how to enhance the mental health of employees should look at different leadership styles.

Finally, this study found a partial mediating effect of meaningful work between empowering leadership and mental health. In order to obtain new results, researchers can incorporate mediators or moderators into the established framework.

## Data availability statement

The original contributions presented in the study are included in the article/supplementary material, further inquiries can be directed to the corresponding author.

## Ethics statement

The studies involving human participants were reviewed and approved by the University of Technology Sarawak Ethics Committee (UTS-EC). The patients/participants provided their written informed consent to participate in this study.

## Author contributions

All authors listed have made a substantial, direct, and intellectual contribution to the work and approved it for publication.
